# Transcriptome analysis reveals the time of the fourth round of genome duplication in common carp (*Cyprinus carpio)*

**DOI:** 10.1186/1471-2164-13-96

**Published:** 2012-03-19

**Authors:** Jin-Tu Wang, Jiong-Tang Li, Xiao-Feng Zhang, Xiao-Wen Sun

**Affiliations:** 1 The Centre for Applied Aquatic Genomics, Chinese Academy of Fishery Sciences, Beijing 100141, China; 2 College of Fisheries and Life Science, Shanghai Ocean University, Shanghai 201306, China; 3 Heilongjiang River Fisheries Research Institute, Chinese Academy of Fishery Sciences, Haerbin 150070, China

**Keywords:** Sequence assembly, Genome duplication, Common carp, Substitution rate, Speciation

## Abstract

**Background:**

Common carp (*Cyprinus carpio*) is thought to have undergone one extra round of genome duplication compared to zebrafish. Transcriptome analysis has been used to study the existence and timing of genome duplication in species for which genome sequences are incomplete. Large-scale transcriptome data for the common carp genome should help reveal the timing of the additional duplication event.

**Results:**

We have sequenced the transcriptome of common carp using 454 pyrosequencing. After assembling the 454 contigs and the published common carp sequences together, we obtained 49,669 contigs and identified genes using homology searches and an ab initio method. We identified 4,651 orthologous pairs between common carp and zebrafish and found 129,984 paralogous pairs within the common carp. An estimation of the synonymous substitution rate in the orthologous pairs indicated that common carp and zebrafish diverged 120 million years ago (MYA). We identified one round of genome duplication in common carp and estimated that it had occurred 5.6 to 11.3 MYA. In zebrafish, no genome duplication event after speciation was observed, suggesting that, compared to zebrafish, common carp had undergone an additional genome duplication event. We annotated the common carp contigs with Gene Ontology terms and KEGG pathways. Compared with zebrafish gene annotations, we found that a set of biological processes and pathways were enriched in common carp.

**Conclusions:**

The assembled contigs helped us to estimate the time of the fourth-round of genome duplication in common carp. The resource that we have built as part of this study will help advance functional genomics and genome annotation studies in the future.

## Background

The *Cyprininae *family includes zebrafish (*Danio rerio*) and the several economically important cultivated carp, such as common carp (*Cyprinus carpio*), grass carp (*Ctenopharyngodon idella*), silver carp (*Hypophthalmichthys molitrix*) and bighead carp (*Hypophthalmichthys nobilis*). Teleosts are widely believed to have gone through an additional round of whole genome duplication referred to as the 3R hypothesis, as compared to mammals. This third round (3R) of whole genome duplication is specific to ray-finned fish and possibly occurred about 360 million years ago (MYA), preceding the divergence of the teleosts [1]. The 3R duplication may have led to the major diversification of the teleosts [2-4]. The chromosome number of common carp (n = 50) is twice that of most other *Cyprinidae*. Thus, it has been assumed that common carp have undergone a fourth round (4R) of genome duplication. Indeed, previous studies have found more copies of several genes and microsatellites in common carp than in most other *Cyprinidae *and various estimates of the time of the additional round of genome duplication have been made. An analysis of the *c-myc *genes in common carp estimated that the tetraploidization event occurred 58 MYA [5] while another study, based on other duplicated genes in common carp, reported a tetraploidization time of less than 16 MYA [6]. Using 59 microsatellites, David et al. [7] estimated that the 4R genome duplication had occurred about 12 MYA. However, these conflicting estimates of the time of genome duplication were all based on small data set. Thus, to obtain a more accurate estimate of the duplication time, it is necessary to use a larger data set.

For species for which the whole genome sequence is not yet available, transcriptome analysis is an alternative method that has been used to discover new genes and to investigate gene expression. A large set of common carp ESTs produced using Sanger sequencing has been developed and used to study traits in common carp [8-11]. More recently, second generation sequencing platforms have been applied to transcriptome sequencing [12-14], making the transcriptome more readily accessible. Transcriptome analysis is a power tool that has been used to study various genome feature, including genome duplication [15,16]. When the synonymous substitution rate (*Ks*) in two paralogous sequences is assumed to increase approximately linearly with age [17], paralogous pairs can be sorted along with their relative ages of duplication by estimating their *Ks*. A genome duplication event would result in a sharp increase in the number of paralogous genes, yielding a secondary peak in the *Ks *distribution of paralogous pairs. Therefore, a secondary peak in the paralogous *Ks *distribution indicates one genome duplication event. Large-scale transcriptome data for the common carp will help the study of the additional genome duplication and improve estimates of the timing of this event in this species.

Here, we have compared the contigs from common carp with zebrafish genes: 1) to assess the relative age of the separation between zebrafish and common carp; 2) to estimate the time of the additional genome duplication event in common carp; and 3) to determine biological processes and pathways enriched in common carp.

## Results

### Hybrid assembly of the common carp 454 contigs and public ESTs/mRNAs

The 454 pyrosequencing generated 242,261 reads, encompassing about 52.9 Mb of sequencing data. The average length of the 454 reads was 218 bp. After the initial adapter trimming and quality filtering, we assembled the remaining 241,170 cleaned reads using Celera assembler [18] and obtained 8,422 contigs and 60,910 singletons. We downloaded 34,067 common carp ESTs and 989 mRNAs from NCBI sequence database. After filtering out possible vector sequences, 33,259 cleaned ESTs and 987 cleaned mRNAs were assembled with 454 contigs/singletons into 51,065 contigs using CAP3 [19]. Further, to avoid redundant gene identification and annotation caused by alternative splicing, we performed an all-against-all BLASTN search on the CAP3 contigs. If the alignments of two sequences had 100% identity over 100 bp, they were considered to be from the same genes as the result of alternative splicing. We selected the longest contigs to represent these genes and finally obtained 49,669 unique contigs. The N50 length of this set of contigs was 654 bp.

### Assessment of hybrid assembly

To assess the quality of our assembly, the CAP3 contigs were compared with 40 published paralogous mRNAs [20 pairs of paralogs, see Additional file 1: Table S1]. The 40 mRNAs were aligned with the CAP3 contigs using BLAT and the CAP3 contig that aligned best with each of the mRNAs was selected. Although the highest identity between these paralogous genes was 97%, all the mRNAs matched distinct CAP3 contigs with the full length sequences covered. This result indicated that our assembly was of high quality.

Without a genome sequence, it is difficult to determine splicing variants in a *de novo *transcriptome assembly [20]. Spliced variants in the dataset can lead to redundant gene prediction and artificial paralogs. Because splicing variants were from the same genes and share common exons, we filtered out possible alternative splicing variants using high sequence identity (100%) over long matched region (> 100 bp). We evaluated the reliability of this strategy using zebrafish transcripts from the Ensembl database [21] as a test dataset. In the Ensembl database, 11,227 zebrafish genes have 30,963 spliced variants and form 36,087 splicing pairs. We identified 31,957 pairs of spliced variants using our strategy; 28,434 of them were in the Ensembl splicing pairs. This result suggested that our strategy had the sensitivity of nearly 80% with a low error rate of only 11% (3,523 out of 31,957).

### Gene prediction from common carp contigs

**Figure 1:**
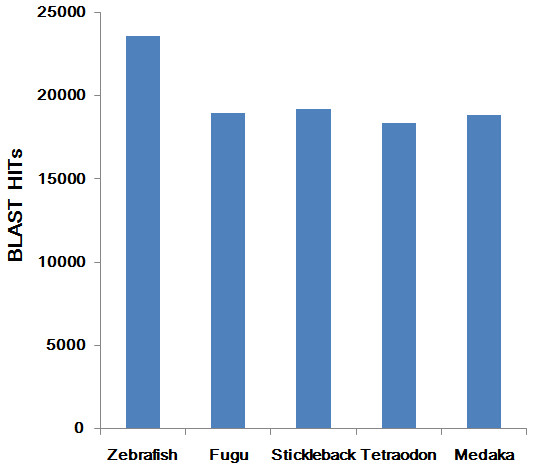
**A bar plot showing the hits to protein sequences from five sequenced teleost species**. Alignments of common carp contigs to protein sequences from Zebrafish, Fugu, Stickleback, *Tetraodon *and Medaka, respectively.

We used homolog searches and an ab initio prediction method to annotate the common carp contigs. By searching against three different protein databases using BLASTX with a cutoff e-value of 1e-5, we found 24,784 contigs that had BLASTX hits to at least one of these databases. Most contigs were homologous to sequences from the fish protein database (Table 1). As shown in Figure 1, the common carp contigs had the greatest number of hits against zebrafish sequences. After running the unmatched contigs against UTRdb [22], a database of 5' and 3' untranslated regions (UTRs) of eukaryotic mRNAs, we found another 3,658 contigs that were homologous to UTRs, indicating that these contigs might represent the UTRs of common carp protein-coding genes. An additional 14,524 contigs could be aligned to the NCBI nonredundant nucleotide database using BLASTN. Since these contigs had hits to known proteins or nucleotide sequences, they might be conserved genes.

**Table 1:** Summary of common carp contig annotation

Methods		Database	Number
Homolog search	Protein-coding	Fish protein database*	24,167
		UniProt database	409
		NCBI nr protein database	208
		UTRdb	3,658
		NCBI nr nucleotide database	14,524
ab initio search	Protein-coding	CPC	47
Unknown			6,656

* Fish protein database consists of protein sequences from Zebrafish, Fugu, Stickleback, *Tetraodon *and Medaka.

Finally, among the remaining unidentified contigs, an ab initio method using the CPC predicted that 47 of the contigs were potentially coding sequences. Because these contigs had no matches to known proteins, they might be common carp specific protein-coding genes. The remaining unknown 6,656 contigs had neither protein-coding potential nor known homologs, indicating that they were probably transcribed from common carp intergenic regions of the common carp genome. The complete annotation statistics are shown in Table 1.

To validate the reliability of the *de novo *assembly and the assembled contigs, gene expression was examined in RNAs from the pooled-tissues by PCR. Twenty contigs, including conserved genes, common carp specific protein-coding genes and unknown contigs [see Additional files 2: Methods S1 and 3: S2], were selected randomly. Primers were designed specifically for the selected contigs to avoid amplifying paralogs. The results showed that all the selected contigs could be amplified [see Additional file 4: Figure S1], indicating that these contigs were correctly assembled and truly expressed.

### Genome speciation event deduced from orthologous pairs between common carp and zebrafish

**Figure 2:**
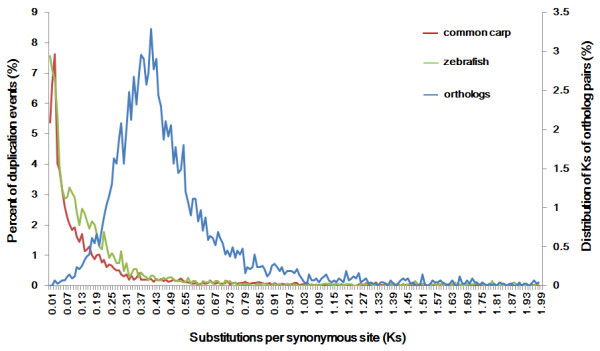
***Ks *value distribution to identify the genome duplication event and the speciation event**. Data was grouped into bins of 0.01 *Ks *units for graphing. For common carp and zebrafish, the *Ks *distributions of duplication events were shown in red and green respectively. A secondary *Ks *peak within common carp indicated the genome duplication (red line). Given the rate of substitutions/synonymous site per year, the peak indicated the time of the 4R of genome duplication. Within zebrafish, no secondary peak in the *Ks *distribution of paralogous sequences was observed (green line). The *Ks *distribution of the orthologous pairs was plotted in blue line and showed a distinct secondary *Ks *peak, indicating the speciation time between these two species.

We assumed that one round of genome duplication occurred in common carp after the speciation while zebrafish had no further genome duplication. A secondary peak in the orthologous *Ks *value distribution indicates a speciation events [16]. Therefore, we firstly estimated the genome speciation time based on the *Ks *distribution of orthologous pairs between the two species. We identified 4,651 orthologous pairs between common carp and zebrafish using the reciprocal best blast hit approach with a stringent e-value cutoff (1e-20) as described previously [16]. The *Ks *distribution of these 4,651 orthologous pairs showed a distinct secondary *Ks *peak at 0.42 (Figure 2). Considering a clock-like rate of synonymous substitution of 3.51 × 10^-9 ^substitutions/synonymous site per year [7,17], the speciation between common carp and zebrafish was estimated to have occurred ~120 MYA. This estimated speciation time is earlier than the time predicted from previous reports based on individual genes [23].

### Genome duplication event estimated from *Ks *distribution of paralogous pairs

A secondary peak in the paralogous *Ks *distribution plot can provide signatures of genome duplication within a species [16]. To evaluate the quality of the paralogs identification by our strategy, we performed an all-against-all BLASTN using the same 20 paralogous pairs. A total of 18 pairs (90% of the real paralogous pairs) satisfied the criteria for paralogous identification [see Additional file 1: Table S1], indicating that the customized parameter was suitable for paralogs identification. We identified 129,984 and 46,385 paralogous pairs within common carp and zebrafish, respectively. These paralogous pairs were then organized into 4,689 and 869 gene families in carp and zebrafish respectively, by a single linkage clustering method. The numbers of sequences in the paralogous pairs and gene families are shown in Table 2 and the *Ks *distribution plots for the two species are shown in Figure 2. We observed an obvious secondary peak in the *Ks *distribution of paralogous sequences within common carp (with a mode at *Ks *= 0.02 to 0.04), indicating a genome duplication event. With the rate of 3.51 × 10^-9 ^substitutions/synonymous site per year [7,17], this duplication was estimated to have occurred 5.6 to 11.3 MYA. This estimate is more recent than previous ones [5-7]. Together with the estimated speciation time, these data suggested that this duplication event occurred long after the speciation. Within zebrafish, no secondary peak in the *Ks *distribution of paralogous sequences was observed, indicating that after speciation no genome duplication event had occurred. Together, these data supported the hypothesis that common carp had undergone one extra round of genome duplication compared with zebrafish.

**Table 2:** Number of sequences and paralogs within common carp and zebrafish

Species	Sequences in final dataset^a^	Paralogous pairs	Paralogous sequences^b^	Percentage of paralogs^c^	Gene families^d^	Duplication Event with median *Ks *< 2^e^
*common carp*	49,669	129,984	19,159	38.6%	4,689	8,190
zebrafish	25,348	46,385	3,774	14.9%	869	2,721

a Number of the longest sequences.

b Number of paralogous sequences found in the final dataset using BLASTN search.

c Percentage of paralogous sequences found in the final dataset.

d Number of gene families constructed with paralogous sequences using single linkage clustering.

e Number of duplication events used in the distributions in Figure 2 and of which median *Ks *rates are < 2.

### Comparison of biological process and pathway enriched in common carp and zebrafish

We determined that a whole genome duplication event occurred 5.6 to 11.3 MYA in the common carp. Since then, re-diploidization would have resulted in a pseudo-tetraploid state and differential evolution or loss of duplicated genes. A biological process and pathway comparison between common carp and zebrafish may provide hints of the possible consequences of whole genome duplication and following re-diploidization.

**Figure 3:**
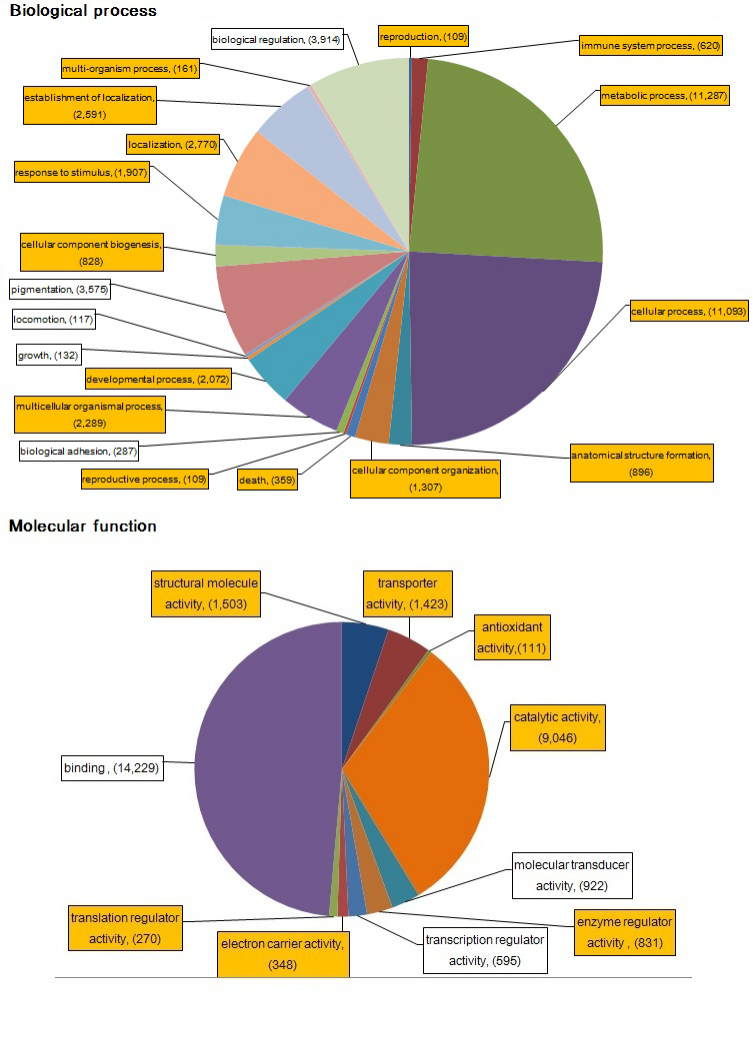
**Distribution of common carp GO terms in biological process and molecular function categories**. The relative proportion of GO terms is represented by more than 100 contigs for the biological process **(A) **and molecular function **(B) **categories in the GO vocabulary. The enriched GO terms in common carp (*p *< 0.05) were highlighted with orange.

We assigned Gene Ontology (GO) terms to 47.2% of the common carp contigs (23,441 out of 49,669) using homologous assignments and Interproscan [24]. This percentage is similar to GO assignments in other fish [25,26]. We then used WEGO to find significantly enriched GO terms in common carp (*p*-value < 0.05) using zebrafish genes as the background. In the biological process category, a total of 15 GO terms (up to 2 level) were significantly overrepresented (*p *< 0.05) while 6 terms were either underrepresented or similar in the common carp relative to the zebrafish GO annotations (Figure 3).

To study the biological pathways involved, we mapped common carp contigs to pathways in the Kyoto Encyclopedia of Genes and Genomes (KEGG) using KOBAS software and a total of 10,308 contigs were mapped to KEGG pathway. Zebrafish genes were also mapped to KEGG pathway and used as background to compare pathway differences between common carp and zebrafish. The statistically enriched pathways (corrected *p-*value < 0.05, Table 3) were consistent with the enriched GO biological process terms. For example, immune-related process, localization and response to stimulus were all indicated in both enriched GO processes and KEGG pathways. These data, therefore, provide an insight into the process and pathway enriched in common carp.

**Table 3:** The enriched pathways in common carp identified by KOBAS (corrected *p*-value < 0.05)

KEGG pathway	ID	Common carp contigs proportion	Zebrafish gene proportion	Biological process GO term (level 2)*
Protein digestion and absorption	ko04974	547/10308	126/7433	multicellular organismal process
Glycolysis/Gluconeogenesis	ko00010	358/10308	76/7433	metabolic process
Pancreatic secretion	ko04972	564/10308	172/7433	Localization
Complement and coagulation cascades	ko04610	360/10308	105/7433	immune system process
Starch and sucrose metabolism	ko00500	243/10308	56/7433	metabolic process
Oxidative phosphorylation	ko00190	415/10308	139/7433	metabolic process
Antigen processing and presentation	ko04612	278/10308	85/7433	immune system process
Pyruvate metabolism	ko00620	175/10308	47/7433	metabolic process
TCA cycle	ko00020	149/10308	36/7433	metabolic process
Pentose phosphate pathway	ko00030	134/10308	31/7433	cellular process
RNA transport	ko03013	352/10308	162/7433	Localization
Mineral absorption	ko04978	134/10308	43/7433	developmental process
PPAR signaling pathway	ko03320	232/10308	96/7433	response to stimulus
Protein processing in endoplasmic reticulum	ko04141	400/10308	215/7433	cellular process
Ribosome biogenesis in eukaryotes	ko03008	113/10308	42/7433	cellular component biogenesis
RNA degradation	ko03018	153/10308	77/7433	metabolic process

* The enriched pathways correspond to biological process GO terms of level 2 in GO vocabulary. These biological processes were still enriched in common carp, indicating the consistence between GO term comparison and KEGG pathway analysis.

## Discussion

Here, we performed transcriptome analysis to estimate the time of the fourth round (4R) of genome duplication in common carp. Earlier, several genes and microsatellites had been used to estimate this. In the present study, we generated an order-of-magnitude more contigs than previously, making the estimated time of the 4R duplication event more reliable.

Timothy et al. [27] reported that the pooled-tissue approach was highly effective in preparing libraries for efficient deep sequencing. RNA that has been pooled from multiple tissues can maximize the number of independent genes for which sequence data can be obtained [27]. Thus, we constructed a pooled-tissue cDNA library and sequenced the library. Although the number of sequencing reads that we obtained were lower than in other studies [28,29], the hybrid assembly built by including public EST/mRNAs improved the sequence coverage and allowed more common carp genes to be identified. The size of common carp genome has been estimated to be 1.7-2.0 Gb [30,31]. Assuming that 1% of the genome encodes mRNAs [26,32], the 49,669 contigs, accounting for 17 Mb, that we obtained provide an estimated coverage of the common carp transcriptome of at least 85%. This estimate is an indication that, in our study, the common carp transcriptome has been sampled broadly with good coverage. Previous studies have suggested that singletons may be biologically valuable [33]. Therefore, we included the singletons in all further analyses. The high coverage of the transcriptome helped in genome duplication detection and function comparison.

Although *de novo *transcriptome assembly can make the genes of non-model organisms with no available genome sequences more readily accessible, a number of challenges including gene duplication or paralogy and alternative splicing still exist. We used stringent parameters for the *de novo *assembly to help differentiate paralogous genes. We then assessed the correctness of the assembly by comparing it with published paralogous genes. These published genes all corresponded to distinct contigs, indicating that the parameters were robust enough to have avoided the creation of chimeric sequences from paralogous transcripts. Spliced variants present in *de novo *assembly dataset are another challenge because their presence can result in redundant gene predictions and paralogs identification. Although spliced variants are difficult to identify without the genome sequences, the strategy that we applied here would have filtered out a high proportion of the alternative variant with a low error rate [see Additional file 5: Figure S2].

To estimate the time of the 4R genome duplication event in the common carp, we estimated the *Ks *values for all orthologous and paralogous pairs. The clear signature of a duplication event within common carp that we obtained showed that the duplication was fairly recent. This is possibly one of the most recent genome duplications in vertebrates. As expected, we did not observe a paralogous peak in zebrafish. These data provided evidence for an additional round of genome duplication in common carp. However, with age increasing, an exponential decrease of density in the distribution of paralogous pairs as a result of the deletion of duplicated sequences would have occurred [34,35]. Because of this effect, common genome duplication events in common carp and zebrafish could not be found.

Zebrafish is the most closely related species to common carp; both belong to the *Cyprinidae *family. The common carp contigs that were generated in this study allowed us to conduct an initial comparative genome analysis between zebrafish and common carp. Because, one more round of genome duplication followed by re-diploidization occurred in the common carp, the comparison of GO biological process terms and KEGG pathways between common carp and zebrafish provided hints of the possible consequences of whole genome duplication and re-diploidization. Interestingly, compared to the zebrafish genes, the common carp contigs were enriched in immune-related terms and pathways, such as, complement and coagulation cascades, and antigen processing and presentation. We examined the functions of the published paralogous genes [see Additional file 1: Table S1] and found that 45% of them (9 pairs) were involved in immune-related pathways. The enriched immune-related contigs might be genes that assist carp in resisting pathogens or might be of help in adapting to different aquaculture growing environment.

## Conclusions

The hybrid assembly of the 454 contigs and published ESTs/mRNAs in this study had significantly expanded the common carp EST resource and provided a valuable dataset for future gene discovery. Our comparative analysis between the common carp contigs and zebrafish genes estimated the time of the 4R of genome duplication event in common carp to be 5.6 to 11.3 MYA and revealed the enriched biological processes and pathways in common carp.

## Methods

### Hybrid assembly of the 454 contigs and public ESTs of common carp

All experimental procedures were conducted in conformity with institutional guidelines for the care and use of laboratory animals in Chinese Academy of Fishery Science, Beijing, China, and conformed to the National Institutes of Health Guide for Care and Use of Laboratory Animals. The animal work was approved by the academic committee in the Centre for Applied Aquatic Genomics (approval ID: 01/2011). Tissue samples were excised from brain, muscle and live of three mature German mirror carps. The tissues were cut into small pieces and immediately pooled into liquid nitrogen. Total RNA in all three tissues was purified using TRIZOL (Invitrogen, Carlsbad, CA, USA) following the manufacturer's instructions. Total RNA quality and quantity were checked by agarose gel electrophoresis containing formaldehyde and using a spectrophotometer. Poly(A) + RNA was purified from total cellular RNA using oligo dT primer. Full-length cDNA was synthesized from 2 μg of poly(A) + RNA using the Clontech SMART cDNA Library Construction Kit (Clontech, Mountain View, CA, USA) according to manufacturer's protocol. The cDNA was amplified using PCR Advantage II Polymerase in 16 thermo cycles with the following thermal profile: 7 s at 95°C, 20 s at 66°C, and 4 mins at 72°C. The amplified cDNA was subsequently purified using the QIAquick PCR Purification Kit (Qiagen, Valencia, CA, USA) to remove fragments of less than 300 bp.

Preparation of the 454 library was performed according to the supplier's instructions (454 Life Sciences, Branford, CT, USA). In summary, approximately 3 μg of amplified cDNA was nebulized and selected for lengths that ranged from 300 to 800 bp. The FLX specific adapters, adapter A (GCCTCCCTCGCGCCATCAG) and adapter B (GCCTTGCCAGCCCGCTCAG), were added to each fragmented cDNA, resulting in adapter A-DNA fragment-adapter B constructs. The DNA fragments were then denatured to generate single-stranded DNA which was then amplified by emulsion PCR for sequencing. The sequencing of the library was performed in one half-plate run on the 454 GS FLX machine. The entire set of reads used for the assembly was submitted to the NCBI Sequence Read Archive under the accession SRA009366 (Submission: SRA009366 by CAFS).

Before assembling the 454 sequencing reads, the adapter sequences were removed. If the ends of one read contained parts of either adapter A (GCCTCCCTCGCGCCATCAG) or adapter B (GCCTTGCCAGCCCGCTCAG), these nucleotides were removed. Next, the Solexa QA package [36] was used to filter out low-quality bases with the following parameters: -probcutoff 0.05 (the quality cutoff score below which the base-calling error was considered to be too high) and -454 (to trim 454 reads). Because homopolymers of poly(A/T) have low quality scores in 454 sequencing [37], this process filters out poly(A/T) sequences. The resulting high-quality (HQ) reads were then assembled using the Celera Assembler 6.1 [18] on a single multiprocessor computer. Most of the parameters were set to the default values; for example, overlapper = mer (a seed and extend overlap algorithm), unitigger = bog (a best overlap graph approach for building unitigs), and doOverlapTrimming = 1 (for overlap-based trimming). To avoid the putative mis-assembly of paralogous genes into chimera contigs, we manually collected 20 pairs of published common carp paralogous genes and analyzed their sequence identity using BLASTN [see Additional file 1: Table S1]. We found that the highest identity between these paralogous gene sequences was 97%. Therefore, the Assembler parameters that might influence sequence assembly were set as: utgErrorRate = 0.029 (the error rate above which the unitigger discards overlaps), ovlErrorRate = 0.029 (overlaps above this limit will not be detected), and cnsErrorRate = 0.029 (he error rate below which consensus finds alignments).

To increase transcriptome coverage, we downloaded 34,067 common carp ESTs from the UniGene database [38] and 989 common carp mRNAs from GenBank. Any vector contamination of the public ESTs/mRNAs was removed using the seqclean program [39]. The 454 contigs/singletons and cleaned ESTs/mRNAs were assembled into contigs using the CAP3 software with default parameters except that we used -p 98 because the highest identity that we found between the carp paralogous genes was 97%.

To avoid the identification of redundant genes as a result of alternative splicing, all-against-all BLASTN searches were performed using CAP3 contigs. If the alignment of two sequences had 100% identity over 100 bps, then they were considered as spliced variants and the longest contigs was selected to represent this gene.

### Gene annotation

To identify the common carp protein-coding genes, we used BLASTX with an e-value of 1e-5 to run our assembled sequences against an in-house fish protein database of protein sequences from Zebrafish, Fugu, Stickleback, *Tetraodon *and Medaka that were downloaded from the Ensembl database [40]. The sequences that had no BLASTX hits to the sequences in the fish protein database were searched against the UniProt database [41] and the NCBI nonredundant protein database [42] using BLASTX with an e-value of 1e-5. Contigs that had no matches to either of these protein databases were aligned against UTRdb [22] because these sequences might represent the UTRs of protein-coding genes. Contigs that had hits to UTR sequences were considered to be protein-coding genes. These remaining unmatched contigs were searched against NCBI nonredundant nucleotide collection using BLASTN with an e-value of 0.05.

Because common carp might have some species-specific protein-coding genes, the contigs that had no matches to any of the known proteins or nucleotide sequences were run through the Coding Potential Calculator (CPC) [43] to predict their coding potential. If CPC predicted that the contig was a coding gene or if it predicted that the contig was non-coding but had an intact open reading frame over 100 amino acids long, then the contig was considered to be a protein-coding gene.

To compare the common carp contigs with the zebrafish genes, we downloaded 45,646 zebrafish protein-coding transcripts from the Ensembl database [40] and selected the longest transcript to represent each gene so as to avoid redundant GO comparison and ortholog identification.

### Estimation of *Ks *in orthologs and paralogs

To identify putative orthologs between common carp and zebrafish, the sequences from common carp and zebrafish were aligned using the reciprocal BLAST (BLASTN) hit method of Blanc et al. [16] with an e-value of 1e-20. Two sequences were defined as orthologs if each of them was the best hit of the other and if the sequences were aligned over 300 bp.

The approach used to estimate the *Ks *of orthologous pairs was adapted from previous studies [15,16]. Briefly, we aligned the common carp contigs to its orthologous zebrafish protein sequence using BLASTX. The longest alignment was selected for analysis. The corresponding common carp sequences were extracted using their aligned coordinates and translated with the *getorf *program from the EMBOSS package [44]. The translated carp amino acid sequences were aligned against the orthologous zebrafish protein using Clustalw [45]. The corresponding codon alignments were produced using PAL2NAL [46]. Finally, *Ks *were estimated using a maximum likelihood method in the CODEML program (runmode-2) of the PAML package [47].

Paralogs within the common carp and zebrafish sequences were identified by all-against-all BLASTN searches. Because of length variations between the sequences, we modified the procedure of Ding et al. [48] and defined two sequences as paralogs if the aligned regions were over 70% of the shorter sequences. To estimate the *Ks *for common carp paralogs, we first aligned the sequence pairs using TBLASTX and then followed the same steps that were used in the *Ks *estimation pipeline in orthologous pairs. Because zebrafish genes have well-annotated protein sequences, the protein sequences of two paralogous genes were aligned with Clustalw [45] and the corresponding codon alignments were produced using PAL2NAL [46]. *Ks *was estimated based on the codon alignments.

### Detection of genome speciation and duplication events

The detection of genome speciation and the duplication event was carried out according to the procedure described by Blanc et al. [16] which were based on the *Ks *distribution of orthologous and paralogous pairs, respectively. For detection, we used only those alignments that were longer than 30 amino acids and had *Ks *< 2 to minimize statistical artifacts caused by short alignments and the saturation of *Ks *[17].

The *Ks *frequencies of orthologous pairs were plotted and the *Ks *peak was used as a potential indicator of the genome speciation event. To detect genome duplication event, the common carp paralogous pairs were organized into gene families according to previous studies [15,16]. One contig was selected to represent one gene cluster, obviating possible redundant *Ks *from multiple entries of the same gene. A gene family of *n *members was assumed to be the results of *n *- 1 gene duplication events. A hierarchical clustering method described previously [16], was used to reconstruct the tentative phylogeny of each gene family. Briefly, in one gene family, all contigs were treated first as a separate cluster. Then, the two clusters (A and B) with the smallest *Ks *values were grouped into a new cluster containing all the contigs. The median *Ks*, obtained for all possible pairs between a contig in cluster A and a contig in cluster B, was used as the *Ks *for the new cluster. Every clustering was assumed to represent one gene duplication event. These steps were repeated until all the contigs were grouped into a single cluster. The obtained *Ks *values that were obtained in each clustering were plotted and the *Ks *peak was assumed to indicate the genome duplication event.

### Biological process and pathway enriched in common carp compared with zebrafish

To study the biological processes enriched in common carp, we annotated each contig by assigning the GO terms associated with the top hit in the fish protein database to the common carp contig. If a contig could not be annotated with a homologous assignment, then Interproscan [24] was used with the default settings to annotate the contigs with GO terms. To study the pathways involved, we used the KOBAS software [49] to map common carp contigs to KEGG pathways based on sequence similarity.

We retrieved the GO terms for the zebrafish protein-coding genes from Ensembl database and kept non-redundant GO terms for every zebrafish gene because redundant GO terms might be assigned to the gene spliced variants. We used WEGO [50] to identify significantly enriched GO terms in the common carp contigs using zebrafish genes as the background. WEGO uses the Pearson Chi-Square test to indicate significant relationships between two input datasets. The biological process terms with *p*-value ≤ 0.05 were considered to be statistically enriched in common carp contigs. To study the enriched pathways by common carp, we assigned KEGG pathways to the zebrafish genes using KOBAS [49] and then used the software to compare the proportion of common carp contigs in each pathway against the proportion of zebrafish genes in the same pathway. KOBAS used fisher-exact test to identify significant pathways and then performed an FDR correction to reduce Type-1 errors. KEGG pathways with corrected *p *values ≤ 0.05 were considered to be statistically enriched in common carp.

## Competing interests

The authors declare that they have no competing interests.

## Authors' contributions

JTW and JTL conducted the bioinformatic analysis and were involved in writing the manuscript. XFZ was responsible for samples preparation and 454 pyrosequencing. JTL and XWS supervised the entire study and provided assistance in manuscript preparation. All authors read and approved the final manuscript.

## Supplementary Material

Additional file 1 **Table S1 Twenty pairs of published common carp paralogs**. Click here for file

Additional file 2 **Methods S1 Sample preparation and PCR validation of selected contigs**. Click here for file

Additional file 3 **Table S2 Primers designed specifically for the selected contigs that we assembled**. Click here for file

Additional file 4 **Figure S1 PCR products of the selected contigs**. Click here for file

Additional file 5 **Figure S2 Spliced variants detected under different alignment lengths using zebrafish variants as a test dataset**. Click here for file
