# Supplementary material for: Transcriptome analysis reveals the time of the fourth round of genome duplication in common carp (Cyprinus carpio)
Source: BMC Genomics. 2012 Mar 19;13:96. doi: 10.1186/1471-2164-13-96 (PMC3352309; doi:10.1186/1471-2164-13-96)
Supplement: Additional file 1 — Table S1 Twenty pairs of published common carp paralogs. [file 1471-2164-13-96-S1.DOC]

| Pairs | Gene Name | Accession | Identity | Sequence Coverage in shorter sequence | Publication |
| --- | --- | --- | --- | --- | --- |
| 1 | gonadotropin type I | M37379 | 84% | 99% | 3246480 |
|  | gonadotropin type II | M37380 |  |  |  |
| 2 | Gh1 | AJ640135 | 95% | 100% | 8025730 |
|  | Gh2 | AJ640136 |  |  |  |
| 3 | cERK1 | AB006038 | 77% | 61% | 9603989 |
|  | cERK2 | AB006039 |  |  |  |
| 4* | C1rs-A | AB042609 | 88% | 86% | 11220628 |
|  | C1rs-B | AB042610 |  |  |  |
| 5* | CfI-A | AB072912 | 83% | 96% | 12618913 |
|  | CfI-B | AB072913 |  |  |  |
| 6* | Mbl1 | AB110825 | 97% | 97% | 17015733 |
|  | Mbl2 | AB110826 |  |  |  |
| 7 | aspolin2-1 | AB189966 | 95% | 100% | Fish. Sci. 71, 904-913 (2005) |
|  | aspolin2-2 | AB189967 |  |  |  |
| 8* | interleukin 1 beta 2-1 | AJ401030 | 97% | 100% | 12946102 |
|  | interleukin 1 beta 2-2 | AJ401031 |  |  |  |
| 9* | NILT1 | AJ811994 | 84% | 47% | 15702329 |
|  | NILT2 | AJ811995 |  |  |  |
| 10 | leptin-II | AJ830744 | 89% | 99% | 16935838 |
|  | leptin-I | AJ830745 |  |  |  |
| 11 | erythropoietin-I | AJ831393 | 94% | 100% | 16614377 |
|  | erythropoietin-II | AJ831394 |  |  |  |
| 12* | ifng2a | AM168523 | 90% | 97% | 18657572 |
|  | ifng2b | AM168524 |  |  |  |
| 13 | Pitx2 I | EF051103 | 96% | 100% | 20850444 |
|  | Pitx2 II | EF051104 |  |  |  |
| 14* | CD8a1 | EU025118 | 83% | 100% | 18022233 |
|  | CD8a2 | EU025119 |  |  |  |
| 15 | cyp19a | EU375455 | 68% | 74% | 18430424 |
|  | cyp19b | EU375456 |  |  |  |
| 16 | FGFR1a1 | EU919569 | 95% | 100% | 19733072 |
|  | FGFR1a2 | EU919570 |  |  |  |
| 17 | gpx4a | FJ656211 | 79% | 85% | 19345279 |
|  | gpx4b | FJ656212 |  |  |  |
| 18* | MyD88a | GU809230 | 92% | 95% | 21087670 |
|  | MyD88b | GU809231 |  |  |  |
| 19 | pro-opiomelanocortin-II | Y14617 | 93% | 99% | 9806347 |
|  | pro-opiomelanocortin-I | Y14618 |  |  |  |
| 20* | CD8b1 | EU025120 | 92% | 98% | 18022233 |
|  | CD8b2 | EU025121 |  |  |  |

*: These paralogs were involved in immune-related processes, indicated by the publication.
