# Supplementary material for: Transcriptome analysis reveals the time of the fourth round of genome duplication in common carp (Cyprinus carpio)
Source: BMC Genomics. 2012 Mar 19;13:96. doi: 10.1186/1471-2164-13-96 (PMC3352309; doi:10.1186/1471-2164-13-96)
Supplement: Additional file 2 — Methods S1 Sample preparation and PCR validation of selected contigs. [file 1471-2164-13-96-S2.DOC]

**Sample preparation**

A total of 3 German mirror carps (average weight: 200g) was obtained from Freshwater Fisheries Research Center of the Chinese Academy of Fishery Sciences, Wuxi, China. Fish were maintained in out-door tanks with running fresh water at 23℃ and fed a commercial diet twice daily. All experimental procedures were conducted in conformity with institutional guidelines for the care and use of laboratory animals in Chinese Academy of Fishery Science, Beijing, China, and conformed to the National Institutes of Health Guide for Care and Use of Laboratory Animals. Fish were anaesthetized with eugenol. Tissues including brain, muscle and liver for RNA extraction were carefully separated and then snap stored at -80℃.

**PCR validation**

Total RNAs of tissues were isolated using TRIzol Reagent (Invitrogen) following the manufacturer’s instructions. Final elution was carried out in nuclease-free water and RNA concentrations were measured by NanoVue Plus Spectrophotometer (GE Healthcare Life Sciences) and the integrity was confirmed by analysis on a 1% agarose gel before cDNA synthesis. The RNAs from the above tissues were mixed together, each with equivalent concentration. Then the RNAs were treated with DNase I (Invitrogen) to remove genomic DNA and the first strand of cDNA was synthesized by reverse transcription using RevertAid™ H Minus First Strand cDNA Synthesis Kit (Fermentas) with an Oligo(dT)18 primer and followed by RNase H (Invitrogen) treatment. cDNA samples were diluted 1:10 in nuclease-free water before using as template in PCR analysis. We randomly selected 20 contigs including conserved genes, common carp specific protein-coding genes and unknown contigs and then used Primer Premier 5.0 software to design primers [see Additional file 3] for use in PCR. PCR reactions were conducted in 20 μl volume containing 1.2μl cDNA (200ng/μl), 1μl forward and reverse mixed primers (10 μM each), 2μl 10xTaq buffer, 1.2μl Mgcl2 (25mM ), 2μl dNTPs (2mM) and 0.5U of Taq DNA polymerase (Fermentas). DNA was amplified on ABI 9700 thermal cycler (Life Technologies) under the following cycling conditions: initial denaturation at 95°C for 5 min; then 35 cycles of 94°C for 30 sec, 53°C for 30 sec and 72°C for 45 sec; final extension at 72°C for 10 min. PCR products were then analyzed using 1.5% agarose gel. Common carp β-actin gene (GenBank Accession M24113.1) was used to validate the reliability of cDNA template. The contigs with positive PCR amplification were considered to be correctly assembled.
