# Supplementary material for: Transcriptome analysis reveals the time of the fourth round of genome duplication in common carp (Cyprinus carpio)
Source: BMC Genomics. 2012 Mar 19;13:96. doi: 10.1186/1471-2164-13-96 (PMC3352309; doi:10.1186/1471-2164-13-96)
Supplement: Additional file 3 — Table S2 Primers designed specifically for the selected contigs that we assembled. [file 1471-2164-13-96-S3.DOC]

| Annotation | Contig ID | Forward primer | Reverse primer |
| --- | --- | --- | --- |
| Common carp specific genes | 000013968 | GCTGCGATGTCAAGGGAG | CAAAGTTGCGAGCGTGGG |
|  | 000020322 | GTGAAGGCAGTTGATGATG | CCAGACGCTTAGCAATGTA |
|  | 000015879 | TGTCTGGAGGGATGTTAC | CTGAAGAAGAATGGCTGA |
|  | 000016485 | GCTGACAGAAAGGGAACA | AGACCGTGAAGCCATAGA |
|  | 000014738 | GAAGTGAGGCAGCCAGAGA | TCGTTGTCGCTTTCAGATT |
| Unknown contigs | 000000831 | TTACATTTAGCGTGAAGAAG | TAAGAATGAGAAAGCGAGT |
|  | 000004126 | TTGTTACGCAGAACCCTT | CTGTCCATTGGCCTTATT |
|  | 000001602 | CAGCCACCTGTACCACTA | GCATACTTCACAAGCCTCA |
|  | 000012756 | GACCAATCAGCAGCCAGAC | GCAAACGAAAGCAAGAACA |
|  | 000020673 | TGAGCCAAGTGGGAGATA | GCATTAGCAAGCGGTTTT |
| Conserved genes | 000021547 | TCTACGAGCCTCTGCCTGTG | TGGTGCCTGATGGGAATGT |
|  | 000000220 | CCACGGAAATCATCACCAT | TAGCAGCATAAACAAGTCCACAG |
|  | 000022932 | ATGAGGGTGAGGAAATAAGA | TCAATACATCACATAGGCTTC |
|  | 000022620 | CAGCGAGATGGTGTAAGTG | ATCCAAACAGGCTAAATGC |
|  | 000022649 | GATGACCGCAAAGTGAAACC | TCTGACTGGCTGGAGAATGAG |
|  | 000027156 | CACTAAACCAGTCCGTCCC | TGTTCGCAATCACATCGTC |
|  | 000013366 | CTGCGTCATTGGCGTGG | TTGCGAGTGGCTGATAGGG |
|  | 000015055 | GAACTCCCGAAGGAACCAC | ATCTGGCACAGCAAATGGT |
|  | 000008893 | CCTCCTCCTTTCCTGTGAT | CCGAGTTGATACTGACTGACC |
|  | 000011874 | ATCCGTTTCGTGCTTTGTT | CCCTGAATGGGTATTGCTT |
| Common carp β-actin | M24113.1 | TGCAAAGCCGGATTCGCTGG | AGTTGGTGACAATACCGTGC |
