# Supplementary material for: Transcriptome analysis reveals the time of the fourth round of genome duplication in common carp (Cyprinus carpio)
Source: BMC Genomics. 2012 Mar 19;13:96. doi: 10.1186/1471-2164-13-96 (PMC3352309; doi:10.1186/1471-2164-13-96)

A: 1-5: common carp specific protein-coding genes; 6-10: unknown contigs; 11: common carp β-actin; M: DL2000 DNA marker.

B: 1-10: conserved genes; 11: common carp β-actin; M: DL2000 DNA marker.


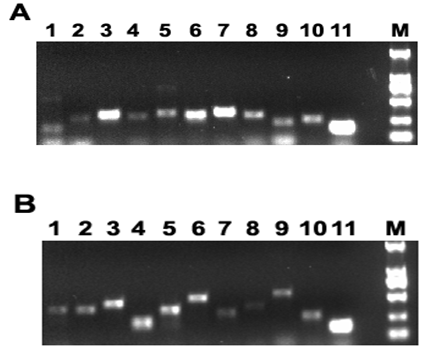

Supplement: Additional file 4 — Figure S1 PCR products of the selected contigs. [file 1471-2164-13-96-S4.DOC]
