# Supplementary material for: Transcriptome analysis reveals the time of the fourth round of genome duplication in common carp (Cyprinus carpio)
Source: BMC Genomics. 2012 Mar 19;13:96. doi: 10.1186/1471-2164-13-96 (PMC3352309; doi:10.1186/1471-2164-13-96)
Supplement: Additional file 5 — Figure S2 Spliced variants detected under different alignment lengths using zebrafish variants as a test dataset. [file 1471-2164-13-96-S5.DOC]

The sensitivity is the proportion of spliced pairs detected by both our strategy and the Ensembl annotation to all spliced pairs in Ensembl. The error rate is calculated as the proportion of spliced pairs detected only by our strategy to all pairs in our annotation. The alignment length of 100bp had a high sensitivity with a low error rate, compared to other alignment lengths.

**
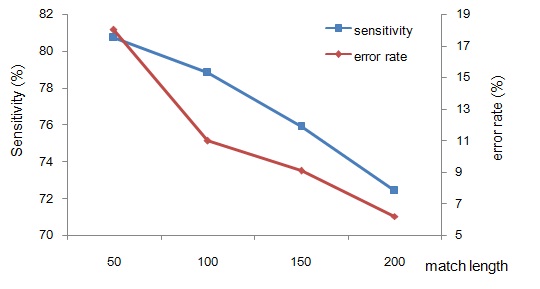
**
